# Elevated Orai1 expression mediates tumor-promoting intracellular Ca^2+^ oscillations in human esophageal squamous cell carcinoma

**DOI:** 10.18632/oncotarget.1903

**Published:** 2014-04-17

**Authors:** Hua Zhu, Hui Zhang, Feng Jin, Mingzhu Fang, Mark Huang, Chung S. Yang, Tong Chen, Liwu Fu, Zui Pan

**Affiliations:** ^1^ Davis Heart and Lung Research Institute, The Ohio State University Wexner Medical Center, Columbus, OH; ^2^ Department of Surgery, The Ohio State University Wexner Medical Center, Columbus, OH; ^3^ Department of Internal Medicine, The Ohio State University Wexner Medical Center, Columbus, OH; ^4^ Sun Yat-Sen University Cancer Center, State Key Laboratory of Oncology in South China, Collaborative Innovation Center of Cancer Medicine, Guangzhou, China; ^5^ Environmental and Occupational Health Sciences Institute; ^6^ Robert Wood Johnson Medical School; ^7^ Department of Chemical Biology, Ernest Mario School of Pharmacy, Rutgers, the State University of New Jersey, Piscataway, NJ

**Keywords:** store-operated calcium entry, STIM1, oncogenic, knockdown, xenograft

## Abstract

Effective treatment as well as prognostic biomarker for malignant esophageal squamous cell carcinoma (ESCC) is urgently needed. The present study was aimed at identifying oncogenic genes involving dysregulated intracellular Ca^2+^ signaling, which is known to function importantly in cellular proliferation and migration. Tumors from patients with ESCC were found to display elevated expression of Orai1, a store-operated Ca^2+^ entry (SOCE) channel, and the high expression of Orai1 was associated with poor overall and recurrence-free survival. In contrast to the quiescent nature of non-tumorigenic epithelial cells, human ESCC cells exhibited strikingly hyperactive in intracellular Ca^2+^ oscillations, which were sensitive to treatments with Orai1 channel blockers and to *orai1* silencing. Moreover, pharmacologic inhibition of Orai1 activity or reduction of Orai1 expression suppressed proliferation and migration of ESCC *in vitro* and slowed tumor formation and growth in *in vivo* xenografted mice. Combined, these findings provide the first evidence to imply Orai1 as a novel biomarker for ESCC prognostic stratification and also highlight Orai1-mediated Ca^2+^ signaling pathway as a potential target for treatment of this deadly disease.

## INTRODUCTION

Esophageal cancer is the 6^th^ leading cause of human cancer death worldwide[1]. Whereas esophageal adenocarcinoma is the main form of esophageal cancer in the United States and other western countries, esophageal squamous cell carcinoma (ESCC) accounts for more than 90% of esophageal cancer cases in such countries as Japan, China, Mongolia and Iran. Because most patients suffering from ESCC are diagnosed at advanced stages of the disease, the 5-year survival rate is poor (19%) and currently represents the fourth poorest among all forms of cancer[2]. Identification of biomarkers for early detection, prognostic stratification, and novel therapeutic interventions are therefore urgently needed for effective management of ESCC.

One emerging exciting area in cancer research is exploration of intracellular Ca^2+^ signaling pathways toward characterization of the molecular mechanisms underlying carcinogenesis and tumor progression. Changes in intracellular Ca^2+^ concentrations ([Ca^2+^]_i_) are well established to influence diverse downstream cellular processes including gene transcription, cell proliferation and migration [3]. In particular, a large body of evidence supports the contribution of altered Ca^2+^ signaling to tumor angiogenesis, progression and metastasis and favors the possibility that genes contributing to such alterations represent novel targets for cancer therapy. Spatially-temporally confined Ca^2+^ signaling is highly regulated in the form of waves, spikes or oscillations [4]. The latter is a remarkable process since its frequency, amplitude and duration can serve as a “calcium code” to activate transcription factors associated with cellular responses to environmental changes [5]. Oscillations in [Ca^2+^]_i_ are coordinated with the release of Ca^2+^ through the InsP3 receptor present on internal Ca^2+^ storage sites such as the endoplasmic reticulum (ER), with the accumulation of Ca^2+^ by the ER, and with Ca^2+^ influx from extracellular Ca^2+^ reservoirs. The last of these processes is governed mainly by a process termed store-operated Ca^2+^ entry (SOCE) [6].

The functional unit of SOCE is currently thought to be comprised of at least two molecular components: Orai1, a Ca^2+^ channel located at the plasma membrane (PM) [7, 8], and STIM1, a Ca^2+^ sensor located at the ER [9, 10]. Upon depletion of ER Ca^2+^ stores, STIM1 molecules cluster at the ER/PM junction such that a retrograde signal is sent to Orai1 for channel opening [11]. Defects in SOCE resulting from genetic mutations in Orail1 or STIM1 have been linked to several human diseases [12], e.g. severe combined immunodeficiency. Furthermore, reduction of STIM1 and/or Orai1 expression by gene silencing techniques decreases growth and metastasis of breast and cervical tumors in mice [13, 14]. Additionally, SOCE has been found to participate the transition from androgen-dependent to androgen-independent stage in prostate cancer [15, 16]. The pathophysiological role of SOCE in tumor growth is further demonstrated by findings with pharmacologic agents. For example, carboxyamidotriazole, a novel anti-cancer drug currently in clinical trials, targets a non-voltage-gated Ca^2+^ channel presumed to be Orai1 [17]. Established SOCE blockers, such as skf-96365 and 2-aminoethyl diphenylborate (2-APB), suppress proliferation of various types of cancer cells [13, 18, 19]. While SOCE has been shown to play an essential role in various cancers, the exact contributions of expression of genes underpinning the SOCE machinery to carcinogenesis and tumor progression remains contradictory and the relevance of Orai1 per se to ESCC in patients are unknown.

The present study was undertaken to reveal the expression profile of SOCE machinery genes in tumor tissues from patients with ESCC and more importantly to explore the clinical significance of Orai1 in esophageal cancer. Using human non-tumorigenic esophageal epithelial and ESCC cell lines, we revealed the alteration in SOCE and intracellular Ca^2+^ signaling in the progression of ESCC. Lastly, we performed functional analysis on Orai1 in ESCC with *in vitro* and *in vivo* approaches. Our attempt is to identify any abnormity in SOCE to be used as diagnostic and/or prognostic biomarker and to provide insights to mechanistic understanding on how such abnormity in SOCE pathway regulates tumor progression.

## RESULTS

### Elevated expression of Orai1 in tumor tissues removed from patients with ESCC

**Figure 1:**
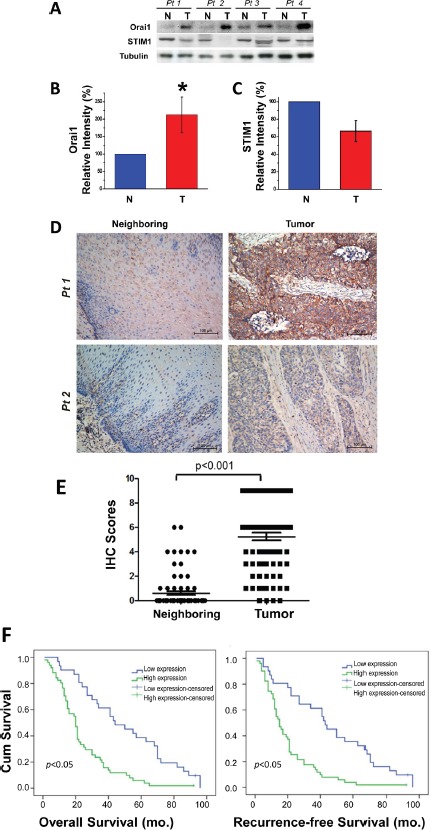
Upregulation of Orai1, but not STIM1, expression in human ESCC tumor tissues and the association between tumor Orai1 expression and prognosis A, representative Western blot images of Orai1 and STIM1 in tumor (T) and neighboring non-tumor (N) tissues removed from patients with ESCC. Tissue lysate (50 μg) were sampled, and tubulin served as the loading marker. B and C, evaluation of Orai1 and STIM1 expression in ESCC tumor tissues as compared to normal neighboring tissues. Densitometry findings for Orai1 and STIM1 were normalized to those for tubulin. Values denote means ± SD (*n* = 34; *p <0.01) using the Student's *t*-test. D, representative immunohistochemistry (IHC) findings for Orai1 expression in human esophageal epithelial tissues. Weak to negative plasma membrane staining of Orai1 was revealed for neighboring non-tumor tissues (left panels) whereas strong to moderate staining was observed in tumor tissues (right panels). Bar indicates 100 μm. E, statistical evaluation of IHC scores for Orai1 expression in normal neighboring and tumor tissues. IHC scores were obtained by multiplying the scores for tumor cell positivity by the scores for Orai1 expression as described in Methods. (Student's *t*-test; *p* <0.001; *n* = 82) F, Kaplan-Meier analyses of overall and recurrence-free survival for ESCC patients with high or low tumor Orai1 expression. The median value of IHC scores was 4; therefore high and low expression scores were defined as scores of ≥4 and <4, respectively. Statistical significance was assessed with the log-rank test. (*p* <0.05; *n* = 82)

Primary tumor specimens and neighboring normal esophageal epithelial tissues were excised from patients with ESCC according to procedures described previously [20]. Quantitative real-time RT-PCR (qRT-PCR) analysis revealed that *stim1* and *orai1* were expressed abundantly in esophageal tumor tissues, but that their homologues (*stim2, orai2* and *orai3*) were less abundant in these tissues (not shown). The major components of SOCE machinery, Orai1 and STIM1, therefore served as the focus for this present study. Values for *orai1* mRNA in ESCC tumors were 256% of those for paired neighboring normal tissues (Supplementary Fig. S1A,=12, p<0.01). Consistent with these observations, Western blot analyses revealed that Orai1 was expressed to significantly higher degrees in ESCC tumors as compared to normal tissues (Fig. 1A). Based on densitometric findings from 34 paired samples, tumor Orai1 values were always greater than those for paired normal neighboring tissues and the change was more than one fold (Fig. 1B,<0.01; Table S1, 23 pairs). In contrast to previously observed increases in STIM1 expression in cervical cancer [14], expression of STIM1 in ESCC tumor tissues did not differ statistically from that in normal esophageal epithelial tissues, and *stim1* mRNA was found to be even reduced in ESCC tumor tissues (Fig. 1C and Supplementary Fig. S1A). The upregulation of Orai1 expression in ESCC tumors was further confirmed by immunohistochemical (IHC) analyses of human ESCC specimens (Fig. 1D and Supplementary Fig. S2.A and B). IHC findings also confirmed localization of Orai1 to the PM in esophageal epithelial tissues. In 72 of 82 paired samples (88%), Orai1 expression was clearly higher in tumor tissues than in neighboring normal tissues. Mean IHC scores for Orai1 in tumors and neighboring normal tissues were 4.8 and 0.7, respectively, showing statistical significant difference (Fig.1E,<0.001).

### Correlation of Orai1 expression with clinicopathological features and prognosis for patients with ESCC

Next, the association between Orai1 expression and clinicopathological features in patients with ESCC was examined. Carcinoma specimens were divided into two groups: those with high and those with low Orai1 expression according to IHC scores using a cut-off value of 4 (the median value). Multivariate analysis (Table 1) revealed that high expression of Orai1 was positively correlated with histological grade (p=0.019), T stage classification (p=0.029), lymph node metastasis (p=0.010) and advanced clinical staging (p=0.012). No correlation was observed between Orai1 expression and other factors such as age, gender, tumor size and location.

**Table 1:** the association between Orai1 expression and clinicopathological parameters in patients with ESCC

Variables	Number	Orai1 expression		p-value^a^
		High	Low	
Age				0.211^a^
<60 years	39	27	12	
≥60 years	43	24	19	
Gender				0.766^a^
Male	62	38	24	
Female	20	13	7	
Tumor location				0.623^a^
Upper	4	3	1	
Middle	53	31	22	
Lower	25	17	8	
Tumor size				0.533^a^
Diameter≥4	38	25	13	
Diameter <4	44	26	18	
Grade				0.019^b^
G1	28	12	16	
G2	29	19	10	
G3	25	20	5	
Stage				0.012 b
I	14	4	10	
II	30	19	11	
III	38	28	10	
TNM classification				
T				0.029^a^
T1/T2	23	10	13	
T3/T4	59	41	18	
N				0.010^a^
N0	38	18	20	
N1/2/3	44	33	11	

a Pearson Chi-Square;

b Kruskal Wallis Test

Survival data were gathered and analyzed from 82 patients for whom samples of both tumor and neighboring normal tissue were available. Among them, 79 (96%) patients died during follow-up. After examining each variable separately for association with survival status, the association between Orai1 expression and survival was analyzed (Fig. 1F and Supplementary Table S2). Kaplan-Meier survival curves revealed correlations between high Orai1 expression and poor overall survival and between high Orai1 expression and recurrence-free survival with statistical significance for both correlations (Fig. 1F, p<0.01). The statistical significance of these correlations was retained with adjustments for several clinical parameters including age, gender, tumor size, tumor grade, lymph node status, T classification and advanced stage (Supplementary Table S2). Using Cox's proportional hazards model, the relative risks (RR) of high Orai1 expression were calculated as 2.683 for overall survival and 2.752 for recurrence-free survival whereas the corresponding RRs of lymph node status N were calculated to be 1.647 for overall survival and 1.604 for disease-free survival (Supplementary Table S3).

### Orai1 expression and SOCE channel activity in ESCC and non-tumorigenic epithelial cells

**Figure 2:**
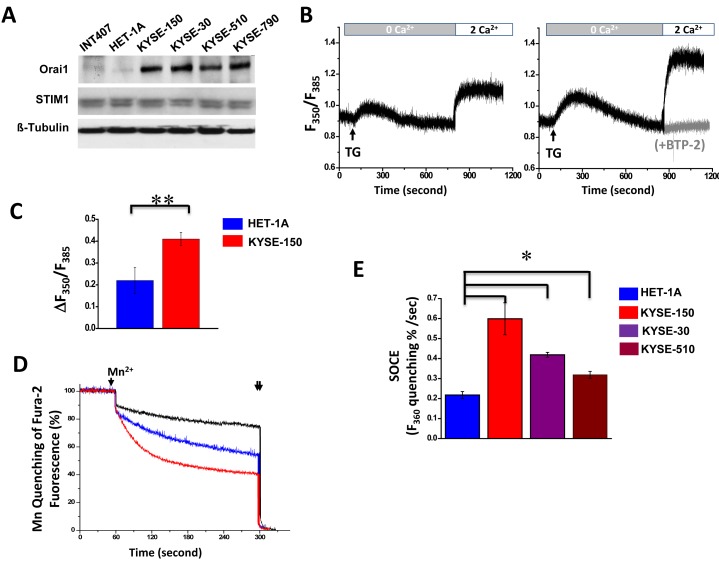
Elevated expression of Orai1 and enhanced SOCE channel activity in human ESCC cells A, western blot findings for Orai1 and STIM1 expression in human ESCC cell lines (KYSE-150, KYSE-30, KYSE-510 and KYSE-790), non-malignant human epithelial cells (INT407 and HET-1A). Tubulin served as the loading control. Images representative of three independent experiments are shown. B, [Ca^2+^]_i_ for Fura 2-loaded HET-1A(left) or KYSE-150(right) cells in BSS as a function of treatment with thapsigargin (TG, 5 μM) and EGTA (0.5mM), or with CaCl_2_ (2 mM). The ratio of F_350_/F_385_ represents [Ca^2+^]_i.._ Gray trace represents KYSE-150 cells treated with BTP-2 (1 μM) for 10 min prior readdition of CaCl_2_. C, statistical evaluation of SOCE in HET-1A and KYSE-150 cells. Δ_350_/F_385_ is used to represent SOCE, which is calculated as the difference between basal and maximal values of F_350_/F_385_ after addition of 2 mM CaCl_2_ in BSS. (n > 5; *, *p* < 0.01.) D, summary of Mn^2+^ entry through SOCE. A Mn^2+^ quenching assay was used as described in Methods. KYSE-150 or HET-1A cells (1x10^6^) were pretreated with TG in BSS containing EGTA to deplete ER Ca^2+^ stores. MnCl_2_ (single arrow) was then added to trigger Mn^2+^ influx through SOCE. Triton X-100 (0.1%, double arrows) was added to permeabilize the cells at the end of protocol. The slope for changes in Fura-2 fluorescence with time was significantly shallower for HET-1A (blue trace) than for KYSE-150 cells (red trace). When KYSE-150 cells were treated with 2-APB (50 M) and MnCl_2_, the slope for changes in Fura-2 fluorescence with time was extremely shallow (black trace). E, statistical evaluation of Mn^2+^ quenching slopes obtained for HET-1A and ESCC cells (KYSE-150, KYSE-30 and KYSE-510). Experiments were performed as described for panel D. Mn^2+^ influx was calculated as the percentage fluorescence decrease per second. Findings are presented as means ± SD from at least 4-6 experiments per condition. (*, *p* < 0.01)

It is well documented that both STIM1 and Orai1 are required for function of SOCE and STIM1 was particularly reported to participate in tumor growth and metastasis in cervical cancer [14]. To elucidate the intriguing function of upregulated Orai1 but not STIM1 in esophageal cancer, the Orai1-mediated SOCE activity were examined in epithelial cell lines derived from human ESCC tumors: KYSE-150, KYSE-190, KYSE-30, KYSE-510 and KYSE-790. Among them, KYSE-150 and KYSE-30 cells have been found highly tumorigenic based on *in vitro* agar assays and *in vivo* tests in nude mice [21]. Two human non-tumorigenic epithelial cell lines (HET-1A, originated from esophagus and INT407, originated from intestine) were included as control [22]. Consistent with findings for human ESCC tumors, expression of Orai1 was found by western blot and quantitative real-time RT-PCR analysis to be elevated in ESCC cells as compared to non-tumorigenic control cells (Fig. 2A and Supplementary Fig. S1B). By contrast, STIM1 was expressed to similar degrees in all non-tumorigenic and cancer cell lines tested (Fig. 2A and Supplementary Fig. S1B). SOCE activity in these cell lines were then examined using a fluorescent Ca^2+^ indicator Fura-2. As shown in Fig. 2B, treatment of 5 μM thapsigargin (TG), an ER Ca^2+^-ATPase inhibitor, resulted in a rapid rise in [Ca^2+^]_i_, consistent with depletion of ER Ca^2+^ stores. Subsequent addition of 2 mM CaCl_2_ to the extracellular bath solution triggered another increase in [Ca^2+^]_i_, consistent with Ca^2+^ influx from the extracellular solution. This event was considered to be attributable to SOCE since an increase was not observed in the presence of skf-96365 (20 μM, not shown), 2-APB (50 μM, not shown) or BTP-2 (1 μM, gray trace of right panel), which are all known SOCE blockers. Δ_350_/F_385_ was calculated to represent the activity of SOCE and the findings showed that SOCE was about twice active in KYSE-150 cells than that in HET-1A cells (Fig. 2B and C). It is worth to note that the expression of STIM1 at a lower level appeared to be still sufficient to support the SOCE activity since knockdown of STIM1 to about one third of original level failed to have impact on SOCE influx (Supplementary Fig. S3). Whereas the basal [Ca^2+^]_i_ showed no significant difference in HET-1A and ESCC cells, the ER Ca^2+^ stores appeared to be significantly less in the former than in the later (Supplementary Fig. S4A and B). Interestingly, the expression level of STIM2 was upregulated in ESCC cells compared with that in HET-1A cells (Supplementary Fig. S4C), which is consistent with the previous reports that STIM2, but not STIM1, regulates basal cytosolic and ER Ca^2+^ levels[23].

Additional Mn-quenching assay was used to compare SOCE activity in HET-1A and ESCC cell lines. Time-dependent quenching of intracellular Fura-2 fluorescence in response to entry of Mn^2+^ via the SOCE mechanism represents a quantitative measure for Orai1 channel activity: the steeper the decline in slope, the greater the channel activity[24, 25]. Compared to that in HET-1A cells (Fig. 2D, blue trace), SOCE activity in KYSE-150 cells (red trace) was significantly increased; the activity in KYSE-150 cells was almost completely eliminated in the presence of 2-APB (50 μM, black trace). Analyses of Mn^2+^ quenching slopes indicated that SOCE activities for all ESCC cells examined were significantly higher than that for HET-1A cells; relative activities were: KYSE-150 > KYSE-30 > KYSE-510 > HET-1A cells (Fig. 2E,<0.05).

### Hyperactivity of intracellular Ca^2+^ oscillations in ESCC cells

**Figure 3:**
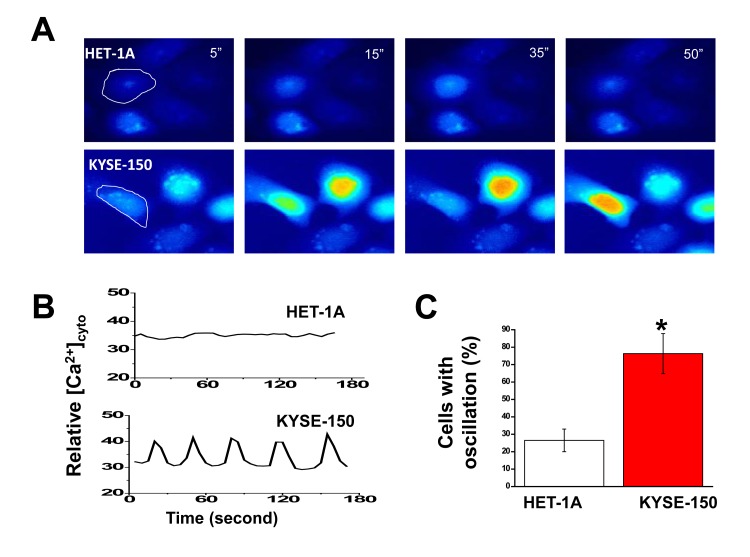
Expression of spontaneous and prominent intracellular Ca oscillations in KYSE-150 cells A, representative live cell images of intracellular Ca^2+^ in HET-1A and KYSE-150 cells loaded with 3 μM Fluo-4-AM as a function of time. The recording was performed in culture condition in the presence of 5% CO_2_ at 37^°^C and under a 20X objective (N.A. 0.75) with the BD Pathway 855 BioImager. B, changes in [Ca^2+^]_i_ over time for the individual cell circled in panel A. C, percentages of cells exhibiting spontaneous Ca^2+^ oscillations within 10 min of recording time. At least 200 of each cell type were examined. (*, *p* <0.01)

To evaluate the impact of increased SOCE on overall intracellular Ca^2+^ homeostasis in ESCC cells, time-lapse imaging was employed using live cells loaded with Fluo-4 AM and maintained in culture medium without phenol red at 37^°^C and in the presence of 5% CO_2_. Whereas HET-1A cells displayed silent nature of Ca^2+^ signaling (Fig. 3 A and B, upper panels and Supplementary Video 1), KYSE-150 cells, interestingly, exhibited spontaneous and prominent intracellular Ca^2+^ oscillations (Fig. 3A and B, lower panels; Supplementary Video 2). The oscillations displayed in sinusoidal form with a frequency of ~0.024 Hz. Within 10 min of recording time, more than 76% of KYSE-150 cells exhibited spontaneous Ca^2+^ oscillations whereas only 26% of HET-1A cells displayed comparable events (Fig. 3C).

**Figure 4:**
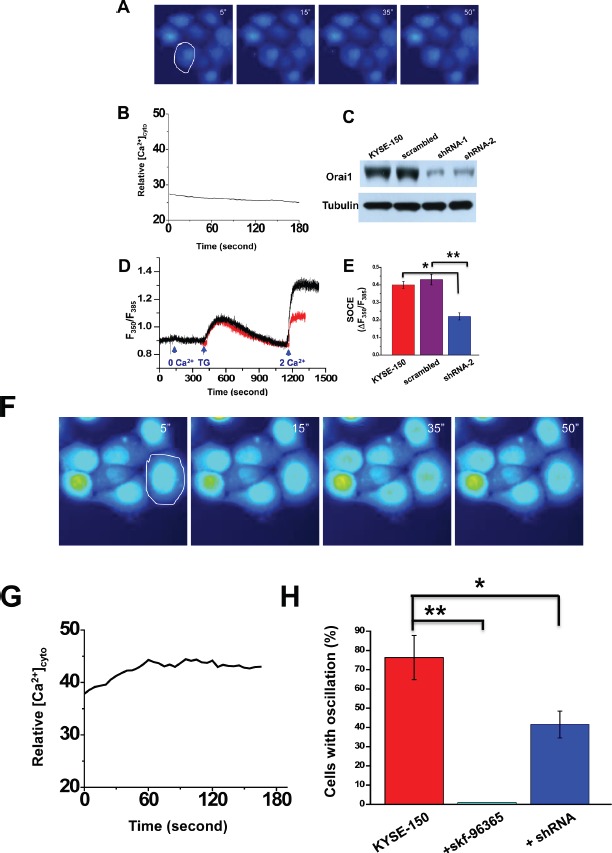
Caoscillations and SOCE in KYSE-150 cells treated with SOCE channel blockers or following knockdown of Orai1 expression A, representative live cell images of [Ca^2+^]_i_ in cells treated with 20 μM skf-96365 as a function of time. Recording conditions were the same as those for Figure 3A. B, relative changes in intracellular Ca^2+^ for the cell indicated in A. C, western blot findings for Orai1 expression in parental cells or cells transfected with plasmids containing a scrambled shRNA sequence (scrambled), anti-Orai1 shRNA-1 (shRNA-1), or anti-Orai1 shRNA-2 (shRNA-2). Tubulin was used to monitor loading. D, [Ca^2+^]_i_ for individual non-transfected (black trace) or shRNA-transfected KYSE-150 cells (red trace) subjected to various treatments. E, analysis of SOCE activity in individual non-transfected KYSE-150 cells or those transfected with shRNA or the scrambled sequence. (*n* >8; *, *p* <0.01; **, *p* <0.01). F, representative live cell images of [Ca^2+^]_i_ as a function of time in cells transfected with anti-Orai1 shRNA. Recording conditions were the same as those described in Figure 3A. G, relative changes in [Ca^2+^]_i_ as a function of time for the individual cell circled in F. H, the percentages of HET-1A or KYSE-150 cells expressing intracellular Ca^2+^ oscillations within 10 min of recording time as a function of no treatment, treatment with skf-96395 (20 μM), or transfection with shRNA. At least 200 cells were examined. (*, *p* <0.01; **, *p* <0.001)

The presence of SOCE inhibitors skf-96365 (20 μM) or 2-APB (50 μM) completely abolished these intracellular Ca^2+^ oscillations in KYSE-150 cells (Fig 4,A and B, and Supplementary Video 3). Addition of Ca^2+^ chelator EGTA (0.5mM) also silenced these oscillations as well.

Evidence that Orai1 is responsible for the spontaneous intracellular Ca^2+^ oscillations displayed in KYSE-150 cells was sought. Several shRNA probes specifically targeting the human *orai1* gene were designed and constructed into the pRiR plasmid, which contains a gene encoding red fluorescent protein (RFP) as a convenient reporter[26]. Transfection with two of these constructs, termed shRNA-1 and shRNA-2, knocked down Orai1 expression in KYSE-150 cells effectively, with ~80% reduction (Fig. 4C). Transfection of pRiR plasmid containing a scrambled shRNA sequence did not change Orai1 expression from that observed in parent cells. This finding indicated that the observed gene silencing effect was not attributable to transfection with plasmid alone and supported use of plasmid containing scrambled sequence as an appropriate control. Both shRNA-1 and shRNA-2 were verified to be specific against the *orai1* gene with no off-target effects since the expression of other Ca^2+^ signaling proteins, i.e. STIM1, SERCA2 and the Na^+^ -Ca^2+^ exchanger (NCX3) were not affected (Supplementary Fig. S5). SOCE activity was then examined as a function of knockdown of Orai1 expression. Treatment with thapsigargin (5 μM) in the presence of EGTA resulted in comparable rapid increases in [Ca^2+^]_i_ in both shRNA-transfected and in non-transfected control cells; subsequent addition of 2 mM Ca^2+^ to the medium triggered a second increase in both preparations but a lesser increase was observed in the transfected preparations (Fig. 4D). Following transfection of KYSE-150 cells with shRNA but not with plasmid containing the scrambled sequence, SOCE activity (Δ_350_/F_385_) was reduced to almost half of that observed in parental cells (Fig. 4E). Intracellular Ca^2+^ signaling as shown in time-lapse imaging was also significantly reduced in KYSE-150 cells transfected with shRNA (Fig. 4, F and G as compared to Fig. 3,A, and B lower panels); less than 40% of these cells exhibited intracellular Ca^2+^ oscillations (Fig. 4H, *p*<0.01). Skf-96365 treatment resulted in almost full suppression of these oscillations, which were present in ~76% of untreated KYSE-150 cells as compared to ~26% of HET-1 cells (Fig. 4H).

### Suppressed cell proliferation, migration and invasion by knockdown of Orai1

Intracellular Ca^2+^ oscillations are thought to stimulate proliferation of lymphocytes through activation of nuclear factor of activated T cells (NFAT) pathway and to promote a variety of other events in cancer cells [27, 28]. However, NFAT was not detected in ESCC cells even after treatment of cyclosporine A to enhance the nuclear translocation of NFAT (not shown). On the other hand, phosphorylated ERK (p44/42) and pAKT (T308) were elevated in KYSE-150 cells compared with those in HET-1A cells and knocking down of Orai1 resulted in decreased phosphorylation forms of ERK and AKT (Supplementary Fig. S6A). The transcription factor MEF2D, a recently identified oncogene in leukemia and liver cancer[29], appeared to be more abundant in ESCC cells than in HET-1A cells (Supplementary Fig. S6B). Such data indicated that ERK, AKT and MEF2D pathways might be the transcription factors involving in Orai1-intracellular Ca^2+^ oscillations-stimulated signaling cascades in ESCC cells.

**Figure 5:**
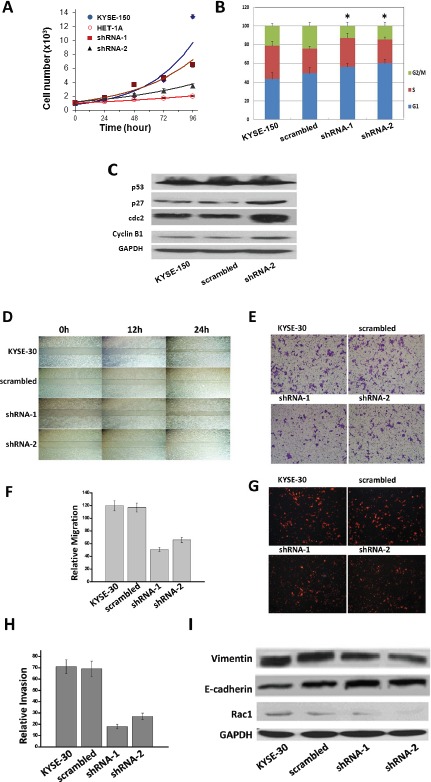
Knockdown of Orai1 suppressed cell proliferation, migration and invasion in ECSS cells A, four cell types were cultured for the times indicated, followed by measurements of cell number. The doubling times for each cell type were: KYSE-150, 22.3 h; HET-1A, 93.5 h; shRNA-1, 40.3 h; shRNA-2, 54.7 h. Results from three independent experiments are presented. (*, *p* <0.05) B, flow-cytometric analyses for KYSE-150 cells transfected with scrambled sequence, shRNA-1, or shRNA-2 (*, *p* <0.05). C, western blot analyses of expression of cell cycle-related proteins in KYSE-150 cells transfected with scrambled sequence, with shRNA-1, or with shRNA-2. GAPDH was used as a loading control. Results are representative of three independent experiments. D, wound-healing capacities. Cells were cultured to confluent monolayers and “wounded” as described in Methods. Images were obtained 0, 12 or 24 h post-wounding. Magnification = 10x. Results are representative of three independent experiments. E and F, migratory capacities. The Boyden Chamber cell migration assay was employed as described in Methods. Photography was performed at 24 h. Three randomly selected fields were viewed and relative migration, the average number of stained cells per field, is shown. Findings from three independent chamber experiments are presented as means ± SEM (**, *p* <0.001). G and H, invasive capacities. The transwell cell invasion assay was performed as described in Methods. Photography was performed at 24 h. Three randomly selected fields were viewed, and relative migration represents the average number of stained cells per field. Findings are presented as means ± SEM from three independent experiments. (**, *p* <0.001). I, western blot findings of Vimentin, E-cadherin and Rac. GAPDH served as the loading control and results are representative of three independent experiments.

The cell proliferation in these cell lines was then compared using MTT assay. The growth of KYSE-150 cells was much faster than that of HET-1A cells, with doubling times of 22.3h comparing 93.5h for the latter (Fig. 5A). Knockdown of Orai1 significantly reduced cell proliferation and increased doubling times (40.3h for shRNA-1 and 54.7h for shRNA-2). Similar results of reduced cell proliferation by reduction of Orai1 were obtained in other ESCC cell lines, such as EC109 cells (not shown). Flow cytometry analyses of cell cycle distribution revealed that reduction of Orai1 resulted in a decrease of cell number in the G2/M phase and an augment in G1 phase compared with parental cells or cells transfected with scrambled shRNA (Fig. 5B & C), indicating delayed G1 to S transition and enhanced G2 to S transition of cell cycle. Next, the changes in several cell cycle related proteins that are known involved in ESCC, were examined. The results shown that knockdown of Orai1 did not change the expression of p53, but resulted in upregulation of cdc2, Cyclin B1 and p27, whereas transfection of scramble shRNA had no effects (Fig. 5D). Given that the G1 to S phase transition is controlled by p27 through inhibition of Cyclin E-CDK2 complex and G2 to M phase transition is governed by cdc2-Cyclin B complex, the Western blot results were consistent with the cell cycle distribution.

The effects of decreased Orai1 expression on ESCC migration and invasion were examined using KYSE-30 cells, a commonly employed metastatic model. The wound-healing assay was selected to measure directional cell migration *in vitro*. Cells were grown to confluence and a “wound” was placed in the middle of the culture plate with a pipette tip. In the absence of serum, parental cells or cells transfected with scrambled shRNA migrated and filled the gap after 24h (Fig.5D, upper panels); by contrast, cells transfected with shRNA-1 or shRNA-2 failed to fill the gap (Fig.5D, lower panels). The Boyden chamber assay was employed to confirm these findings. This assay permits cells to migrate through an 8 μm-pore membrane at the bottom of the chamber; cells that have migrated through the pore are visualized by crystal violet staining. As shown in Fig. 5, E and F, fewer stained cells were observed following transfection of the cells with shRNA-1 or shRNA-2 as compared to parental cells or those transfected with the scrambled sequence. The number of migratory cells was decreased by approximately half as a consequence of transfection with shRNA-1 or shRNA-2 but not as a consequence of transfection with the scrambled sequence (Fig. 5F).

The cell invasion was then examined using the same Boyden chamber system but with a coating of extracellular Matrigel applied to cover the membrane. Cells that invaded the matrix were fixed and stained with propidium iodide. Fewer stained cells were observed following transfection of the cells with shRNA or shRNA-2 as compared to parental cells or those transfected with the scrambled sequence (Fig. 5G), and the number of invasive cells was decreased by approximately 70% as a consequence of transfection with shRNA-1 or shRNA-2 but not as a consequence of transfection with the scrambled sequence (Fig. 5H).

It was considered of importance to ascertain whether knockdown of Orai1 affected the expression of proteins involved in migration and invasion. To this end, three proteins central to these processes were selected [30]. Vimentin, an intermediate filament protein, is an important marker of epithelial-to-mesenchymal transition and a requisite regulator of mesenchymal cell migration. E-cadherin function is frequently lost during tumor progression and the transition to a more motile and invasive phenotype. Rac1, a key GTPase, facilitates cell migration in esophageal cancer cells. Transfection with shRNA-1 or shRNA-2, but not with scrambled sequence, resulted in decreased expression of vimentin and Rac1 and up-regulation of E-cadherin (Fig. 5I).

### Prevented tumor growth by blocking SOCE or knockdown Orai1 in vivo

**Figure 6:**
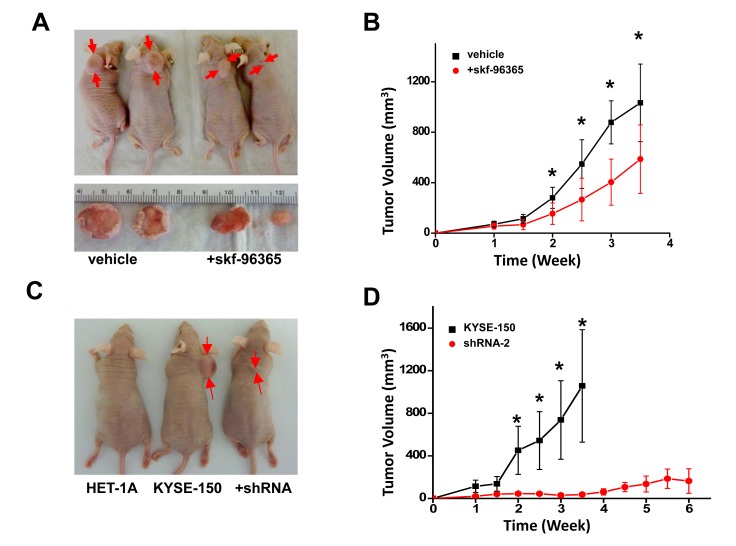
Reduction of SOCE by channel blocker or knockdown of Orai1 prevented tumor growth in xenograft nude mice A, tumors in male NCR nu/nu mice inoculated subcutaneously with 1 × 10^6^ KYSE-150 cells and treated with vehicle or skf-96365 (10 μg/g body weight). Drug or vehicle (DMSO/saline) was administered three times weekly by intraperitoneal injection. Tumors were photographed at 3 weeks post-inoculation. B, tumor volumes for animals described in panel A as a function of treatment time. The black trace indicates vehicle treatment, and the red trace indicates skf-96365 treatment. (*n* = 8 for each group; *, *p* < 0.01) C, tumors in male NCR nu/nu mice inoculated subcutaneously with 1 × 10^6^ HET-1A, non-transfected KYSE-150, or shRNA-transfected KYSE-150 cells. Tumors were photographed at 3 weeks post-inoculation. D, tumor volumes for animals described in panel C as a function of time. The black trace indicates non-transfected KYSE-150 cells, and the red trace indicates shRNA-transfected KYSE-150 cells(*n* = 8; *, *p* < 0.01).

Studies of Orai1 function in cultured ESCC cells were extended to a xenograft animal model. After subcutaneous inoculation of KYSE-150 cells into NCr nu/nu nude mice (5-6 weeks of age), solid tumors formed rapidly (Fig 6, A-D). By contrast, no visible tumor masses were observed throughout the 12 weeks following inoculations with HET-1A cells (Fig 6,C). Intraperitoneal injection of skf-96365 (10 μg/g body weight) every other day for 2 weeks significantly retarded tumor growth (Fig. 6,A and B; *n* = 8, *p* <0.01). The application of skf-96365 at this dose daily for up to two weeks appeared to be safe for these animals since no obvious abnormality or body weight was observed (not shown).

It was considered essential to verify that the high expression of Orai1 displayed by ESCC cells was important for their ability to produce rapidly-growing tumors in these animals. To this end, the nude mice were subcutaneously inoculated with same number of KYSE-150 control cells or cells stably expressing shRNA-2; volumes of tumors generated in these xenograft mice were then measured over 4-6 weeks. As shown in Fig. 6,C and D, volumes of tumors generated from KYSE-150 cells containing shRNA-2 were significantly smaller than volumes of tumors generated from non-transfected KYSE-150 cells. Furthermore, at 6 weeks following inoculations, 3 out of 8 animals inoculated with KYSE-150 cells containing shRNA-2 failed to show a visible tumor. These findings clearly demonstrated that the reduction of Orai1 expression inhibited ESCC tumor growth in xenograft nude mice.

## DISCUSSION

The present report is the first, to our knowledge, to reveal an oncogenic role for Orai1 in esophageal squamous cell carcinoma, a major cause of cancer death worldwide. Compared with that in neighboring non-tumorous esophageal tissues, expression of Orai1 in tumors obtained from patients with ESCC was significantly elevated (Figs. 1A, 1B and 1D). High Orai1 expression was strongly associated with the recurrence rate for this disease independently of other variables and using either overall survival or recurrence-free survival as the endpoint (Fig. 1F and Table S2 and S3). In cultured epithelial cells derived from ESCC patients, strikingly hyperactive intracellular Ca^2+^ oscillations were observed (Fig. 3); reduction of Orail1 function using either pharmacologic or molecular approaches suppressed these oscillations (Fig. 4) indicating that they were mediated by Orai1 channel activity. Moreover, inhibition of Orai1-mediated SOCE by pharmacologic antagonists of the channel or reduction of Orai1 expression by *orai1* knockdown impeded the proliferation and migration of ESCC cells in culture, reduced their capacity for invasion, and altered their expression of proteins intimately concerned with migration and invasion (Figs. 5). Finally, inhibition of Orai1-mediated SOCE by pharmacological antagonists of channel activity or reduction of Orai1 expression in ESCC cells via *orai1* knockdown suppressed the growth of human ESCC tumors in xenografted nude mice (Fig.6). As such, this present study provides the first evidence, to our knowledge, in support of an association between Orai1 expression and the clinical outcome of cancer patients and the hyperactivity of Orai1-SOCE-intracellular Ca^2+^ oscillations pathway in cancer cells.

Similar molecular and pharmacologic approaches employed in this study have been utilized by other investigators to evaluate the importance of STIM1 to the migration and metastasis of breast and cervical cancers. For example, STIM1 was reported to promote cell proliferation and migration, to favor the development of angiogenesis in cervical cancer, and to enhance focal adhesion turnover in breast cancer cells [13, 14, 31]. Chen *et al*, who examined tumor tissues from subjects with early-stage cervical cancer, observed that STIM1 was overexpressed in 71% of these tissues [14]. In the present study, however, expression of STIM1 in tumor tissues from patients with ESCC either did not differ or was reduced as compared to that in neighboring normal esophageal tissues (Fig. 1,). These contradictory findings imply tissue and cancer stage variations in the features of SOCE and/or in the expression of the components of SOCE machinery. Findings from other independent studies are cited here to support this hypothesis. Firstly, down-regulation of SOCE activation and reduction of Orai1 expression were observed by this laboratory and others in prostate cancer cells representing advanced stage [15, 16]. Secondly, Feng *et al.* identified a signaling pathway in which formation of an Orai1-SPCA2 complex elicits constitutive store-independent Ca^2+^ signaling and promotes tumorigenesis in breast cancer[32]. Thirdly, the native SOCE pathway was found to be mediated by Orai3 in estrogen receptor-positive breast cancer cells whereas the canonical STIM1/Orai1 pathway was shown to be used by estrogen receptor-negative breast cancer cells[33]. Lastly, Chantome *et al*. found that knockdown of STIM1 had no effect whereas knockdown of Orai1 inhibited migration of breast cancer cells, indicating STIM1 might not be involved in the metastatic process[34]. The observation of significant higher expression of STIM2 in ESCC cell lines than that in HET-1A cells implies that STIM2 may play an important role in regulation of Orai1 channel activity and overall intracellular Ca^2+^ signaling in this type of cancer. Further investigations of expression of the components of the SOCE machinery and of the functions of the channel during carcinogenesis and tumorigenesis are needed to define the mechanisms through which the process of SOCE is regulated in different cancers.

A proper stoichiometric ratio of Orai1 to STIM1 is required for optimal SOCE function. Overexpression of Orai1 alone has been found to decrease SOCE in some cell types including HEK-293, HeLa and A549 [35]. Recent crystallization study revealed a hexametric structure for the functional Orai channel through coupling with STIM1[36], and provided evidence to support the model that the optimal Orai1/STIM1 for maximal SOCE activation is 2:1[37]. However, variations in the ratios of Orai1 expression to STIM1 expression and in the functional properties of SOCE are reported for different cell types[38]. Any perturbation in the stoichiometry may have significant consequence on SOCE properties and intracellular Ca^2+^ oscillations, and likely tumor progression. Any perturbation in stoichiometry may have significant consequences for the properties of the channel, for the manifestation of intracellular Ca^2+^ oscillations, and for tumor progression. An unexpected finding in the present study was the observed increase in Orai1 expression with no increase in STIM1 expression in ESCC tumor tissues (Fig. 1, and Fig. S1). In addition, knockdown of STIM1 in KYSE-150 cells appeared to have no effect on SOCE activity (Supplementary Fig. S3). These findings support two possibilities: 1) the expression of STIM1 is so abundant in esophageal epithelial cells and thus it is sufficient to cope with the regulatory needs even in the case of significant elevated expression of Orai1 in esophageal cancer cells; 2) STIM1 is not sufficient to support the regulatory needs so that some Orai1 channels may escape coupling with STIM1. Further studies will warrant the differentiation of the two mechanisms. While the present report is in preparation, Wang *et al* identified distinct Orai1-coupling domains in STIM1 and STIM2, which determines the efficacy of coupling between STIM2 and Orai1 as well as whether it contains store-dependent agonist properties[39]. And our data showed that the expression of STIM2 was elevated in ESCC cells compared to that in HET-1A cells. It applauses the possibility that STIM2 may replace STIM1 to couple with Orai1 in cancer cells. Clearly, more studies are urgent to address these following important questions: 1) is uncoupling regulated in response to different cellular requirements in cancer cells and how does regulation occur? 2) are additional factors required for coupling of STIMs with Orai1 and/or for regulation of Orai1 channel gating in ESCC as compared to normal esophageal epithelial cells? 3) does Orai1-STIM2 complex control intracellular Ca^2+^ oscillations and tumor progression in ESCC?

Intracellular Ca^2+^ oscillations are recognized to serve as “calcium code” to initiate many biological processes, such as T-cell proliferation[27]. However, the mechanisms through which oscillatory changes in [Ca^2+^]_i_ are initiated in various cancer cells remain to be defined. It is worthwhile to note that the remarkable prominent intracellular Ca^2+^ oscillations observed in the present study in ESCC cells was recorded for cells in culture medium containing fetal bovine serum (5%) Under this condition, growth factors capable of binding to their specific receptors to stimulate InsP3 receptor-dependent release of Ca^2+^ from the ER were present. Such growth factors may generate partial depletion in ER Ca^2+^ stores and have promoted activation of SOCE in this study. It is interesting to speculate that a particular growth factor receptor is abundantly expressed, or even over-expressed, in ESCC cells and that occupation of this receptor mediated optimal activation of SOCE. For example, epidermal growth factor receptor (EGFR) has been reported to be overexpressed in many cancer cells including ESCC[40]. Activation of this receptor by EGF, by angiotensin II, or by endothelin-1 is known to evoke Ca^2+^ release from ER Ca^2+^ stores. Whether occupation of the EGFR by an appropriate agonist serves to promote full SOCE activation with amplified intracellular Ca^2+^ oscillations in cultured ESCC cells remains to be shown. Further studies of the mechanisms through which the Orai1 channel of cultured ESCC cells is activated are warranted in order to approach an understanding of the nature of ESCC tumor growth *in vivo*.

In summary, this study demonstrated that tumor tissues from patients with ESCC express Orai1 at high concentrations; expression of the channel correlates closely with the recurrence rate for this disease independently of other factors. Orai1 is therefore proposed as an effective biomarker of the prognosis for patients with this form of cancer. A salient property of isolated human ESCC cells is their capacity to exhibit prominent and spontaneous intracellular Ca^2+^ oscillations which depend on Orai1-mediated SOCE. More importantly, evidence provided in this report supports the proposal that increased Orai1-SOCE-intracellular Ca^2+^ oscillations serve to signal activation of downstream pathways that stimulate the proliferation and migration of ESCC cells, enhance their capacity to invade other tissues, and promote ESCC tumor formation and growth *in vitro* and *in vivo*. Findings of this report should greatly facilitate further investigations of the mechanisms underlying the regulation of Orai1 channels in cancer cells and thereby serve to identify targets for novel therapeutic treatments of esophageal cancer.

## MATERIALS AND METHODS

### Patients and tissue specimens

All protocols concerning human subjects were approved by the Regional Ethical Committee of Sun Yat-Sen University, and all investigations with human subjects were conducted after informed consent was obtained. Primary tumors and their neighboring non-tumorous tissues were obtained from 82 patients with ESCC, each of whom underwent surgical resection without preoperative systemic chemotherapy at the Cancer Center of Sun Yat-Sen University. The specimens were collected immediately after surgical removal and fixed with 10% formalin followed by paraffin-embedding. Clinical information was obtained from pathology reports and is summarized in Table S2 and Table 1. The median follow-up period was 25 months (range: 1–96months). Tumor histology confirmed that all specimens were ESCC and that 53 of 82 (64.6%) tumors originated in the thorax. For Western blot and quantitative RT-PCR expression studies, 34 pairs of fresh samples were frozen in liquid nitrogen immediately after surgical removal and maintained at −80^°^C until use.

### Cell lines and cell culture

HET-1A cells were maintained in serum-free LHC-9 medium whereas KYSE-150, KYSE-30, KYSE-510, KYSE-790 and other ESCC cell lines were cultured in RPMI-1640/Ham's F12 (1:1) medium supplemented with 5% FBS[20]. All cell lines were cultured in the presence of 1% penicillin/streptomycin at 37^°^C in a humidified 5% CO2 incubator. HET-1A cells were cultured with RPMI-1640/Ham's F12 (1:1) medium supplemented with 5% FBS for 7 days prior to initiating experiments.

### Plasmids and shRNAs

Multiple short hairpin RNA (shRNA) probes targeting the human *orai1* gene and a probe containing a scrambled sequence (control) were designed and constructed into a pU6-mRFP expression vector as described previously [26]. Two shRNA Orai1 probes, one containing the sequence 5'-cgtgcacaatctcaactcg-3' in the coding region, and another with the sequence 5'- gcactttgaaactgtcctcta-3' in the 3'-UTR region, were verified by Western blot analysis to be effective in silencing the *orai1* gene.

### Western blot analysis

Frozen tissues or cell pellets were treated with RIPA buffer (150 mM NaCl, 50 mM Tris-Cl, 1 mM EGTA, 1% Triton X-100, 0.1% SDS and 1% sodium deoxy cholate, pH 8.0) in the presence of a protease inhibitors cocktail (Sigma). Equal amounts of total protein (25 μg or otherwise stated) were subject to SDS-PAGE (8%). Antibodies used in this study included the following: mouse anti-STIM1 mAb (1:1000, BD Transduction Laboratories, Clone 44); rabbit anti-Orai1 pAb (1:1500, Millipore, against residues 22-40 of the human protein); anti-Tubulin pAb (1:1000, Abcam, against residues 1-100 of the human protein); mouse anti–E-catenin (1:4000, Sigma); rabbit anti-Cdc2 mAb (1:1000, Abcam, #ab32384); mouse anti-Vimentin mAb (1:500, Millipore, # CBL202); anti-cyclinB1 mAb (1:1000, Abcam, #ab32053); anti-p53 mAb (1:1000, Santa.Cruz); rabbit anti-p27 pAb (1:1000, Cell Signaling Technology); rabbit anti-Rac1 pAb (1:500, Proteintech); mouse anti–ß-Actin (1:4000; Sigma); and horseradish peroxidase-conjugated secondary antibodies (Pierce Biotechnology, Rockford, IL). The chemiluminescence of proteins transferred to PVDF membranes was detected with ECL Plus (GE Healthcare Amersham, Piscataway, NJ). Relative protein expression values were quantitatively determined via densitometry with ImageJ software.

### Immunohistochemistry and scoring of Orai1 expression in human tumor tissues

Paraffin-embedded, formalin fixed esophageal cancer tissue sections (4 μm in thick) were deparaffinized with xylene and rehydrated in ethanol. Endogenous peroxidase activity was blocked with 3% hydrogen peroxide (H_2_O_2_) for 30 min at room temperature. Antigen retrieval was performed by pressure cooking sections for 3 min in buffered EDTA solution (pH = 8.0). Sections were treated with 10% normal goat serum at room temperature for 30 min to reduce nonspecific reactions. Antibody against human Orai1 (1:100) was then applied and samples were stored at 4°C overnight in a moist chamber. Samples were treated with secondary antibody (Envision; Dako, Glostrup, Denmark) for 1 h at room temperature and stained with DAB (3,3-diaminobenzidine). Finally, the sections were counterstained with Mayer's hematoxylin, dehydrated, and mounted. A negative control was obtained by replacing the primary antibody with a normal murine IgG. The number of positively stained cells was averaged by two independent investigators using microscopy.

Stained cell proportions were scored as follows: 0 (no stained cell); 1 (10–25% positively stained cells); 2 (26–50% positively stained cells); 3 (51–100% positively stained cells). Staining intensity was graded according to the following standard: 0 (no staining); 1 (weak staining= light yellow); 2 (moderate staining = yellow brown) and 3 (strong staining = brown). The product of [positively stained cell proportion × stained intensity] served as the receptor score. The median value of IHC scores was 4; therefore low and high expression was set at scores of <4 and ≥4, respectively[41].

### Measurement of cell proliferation and cell cycle distribution

Cell proliferation was examined using the standard MTT assay. Cells were seeded at a density of 400 per well in growth medium in 96-well plates and incubated for varying time periods. The number of viable cells at each incubation time was determined by the absorbance of water-insoluble formazan at 570nm using a FlexStation 3 (Molecular Devices, CA).

Flow cytometric analysis was performed with cells that had been seeded at a density of 3×10^5^/well in 6-well plates and then cultured for 72 h in growth medium. Cells were fixed in 70% ethanol/10% PBS treated with 50μg/ml DNase-free RNase; stained for 30 min at 37°C with propidium iodide (50μg/ml) in 0.1% sodium citrate containing 0.1% Triton X-100 and subject to flow cytometry using a FC500 flow cytometer (Beckman Coulter, USA). CellQuest software (BD Biosciences) was used to determine the percentages of cells in the G0/G1, S, and G2/M phases.

### Measurements of intracellular Ca^2+^ concentration and SOCE

Cultured cells were loaded with 5μM Fura-2 acetoxymethyl ester (Invitrogen, OR) for 45 min at 37 ^°^C following a previously published procedure [25]. [Ca^2+^]_i_ was monitored by fluorescence microscopy with a 40x objective (Nikon TE200 Super Fluo, N.A. 1.3) in a dual-wavelength spectrofluorometer (Photon Technology International, Monmouth Junction, NJ). The dual excitation wavelengths were 350nm and 385 nm, respectively; the emission wavelength was 510 nm. ER Ca^2+^ stores were depleted by treatment with 5 μM thapsigargin in BSS solution (in mM: 140 NaCl, 2.8 KCl, 2 MgCl2, 10 HEPES, pH 7.2) containing 0.5 mM EGTA. Ca^2+^ entry was accomplished by addition of CaCl_2_ (2mM). SOCE activity is presented as ΔF_350_/F_385_, the difference between basal and maximal values of F_350_/F_385_ after addition of 2mM CaCl_2_ in BSS solution. Alternatively, SOCE was measured through use of Mn^2+^ quenching assay[25], which is based on measurement of the fluorescence of fura-2 at a Ca^2+^ concentration-insensitive isosbestic point (F_360nm_). Briefly, KYSE-150 or HET-1A cells (1x10^6^) were placed in quartz cuvettes and pretreated with thapsigargin (5μM) in BSS to deplete ER Ca^2+^ stores. Addition of MnCl_2_ (0.5 mM) promoted Mn^2+^ influx through SOCE. At the end of each experiment, Triton X-100 (0.1%) was added to permeabilize the cells for normalization. The slope of changes in normalized Fura-2 fluorescence versus time was calculated as the fluorescence decrease in %/sec.

To record intracellular Ca^2+^ oscillations, cells cultured in 96-well imaging plates (BD Falcon, NJ) were loaded with Fluo-4-AM (3 M) for 20 min at 37^°^C in culture medium without serum. After removal of the fluorescent dye, intracellular fluorescence was monitored under a 20X objective (NA 0.75) using the BD Pathway 855 BioImager system at 37^°^C in the presence of 5% CO_2_ and normal culture medium without phenol red. Time-lapse live cell imaging was recorded and findings were analyzed using either BD Pathway software or ImageJ.

### Cell migration and cellular invasion assays

KYSE-30 cells cultured to confluence in 6-well plates were depleted of serum for 12 h. A wound was then placed by scraping a conventional pipette tip across the monolayer, followed by three washes with PBS. Cell migration and wound closure images were obtained by photography at 0, 12 or 24 h after wounding. Experiments were carried out in triplicate, and identical findings were obtained on at least three occasions.

Alternatively, cell migration was also examined in Boyden chamber system. KYSE-30 cells (5×10^4^) suspended in serum-free medium were added to the upper chamber (6.5-mm diameter, 8-μm pore size, Corning), and the chamber was placed in 24-well dishes. Migration was permitted for 24 h, and cells were then fixed with 4% formaldehyde. Cells that had migrated were stained with crystal violet, and non-migrating cells on upper side of the insert were removed with a cotton swab. Three randomly selected fields (10× objective) were viewed by microscopy and relative migration was determined as the average number of stained cells per field.

Cell invasion assays were conducted using a Boyden chamber but with Matrigel-coated invasion inserts (BD Biosciences, 8 μm pore membranes) placed according to the manufacture's instructions. Transmigrating cells on the underside of the inserts were fixed with 4% formaldehyde and stained with propidium iodide. Fluorescent images of three random fields were acquired the average number of cells per field was calculated and is presented as relative invasion.

### Xenograft assays

Animal care and experiments were approved by the Institutional Animal Care and Utilization Committee (IACUC). In total, 1 × 10^6^ cells in 100μL PBS were mixed with an equal volume of Matrigel (BD Bioscience, San Jose, CA) and then subcutaneously injected into the backs of male NCr nu/nu nude mice (Taconic Farm, NY). For the pharmacological study, total 16 inoculated mice were randomly assigned to two groups with each group containing 8 animals: control and treatment groups. One week after inoculation at that time the tumors were already visible in all animals, the treatment group received intraperitoneal injection of skf-96365 (10 μg/g body weight) every other day for 2 weeks. The control animals were injected with same amount of PBS containing same concentration of DMSO (solvent for skf-96365).

Tumor volumes were evaluated twice weekly after initial detection. Tumor sizes were measured with a digital caliper. The tumor volume in mm^3^ was calculated by the formula: volume = (width) ^2^ × length × 3.14/6. When tumor size reached 15 mm in diameter, animals were euthanized following IACUC guideline.

### Statistical analyses

If not otherwise stated, findings were analyzed with statistical software package SPSS 16.0 and Graphpad Prism 5. The Pearson Chi-square test was used to analyze the relationship between Orai1 expression and various clinicopathological features. Survival curves were generated according to the Kaplan-Meier method and statistical analysis was performed using the Log-rank test. The Cox proportional hazards regression model was used to identify independent prognostic factors. The Student's *t*-test was used to analyze data obtained from investigations of Orai1 expression in human cancer tissues and adjacent non-tumor tissues and from studies of cell proliferation, migration and invasion. Findings from measurements of SOCE and oscillations in [Ca^2^+]i were analyzed using Origin Pro7.0. A *p* value <0.05 was considered statistically significant.

## SUPPLEMENTARY INFORMATION AND FIGURES










