# Supplementary material for: Elevated Orai1 expression mediates tumor-promoting intracellular Ca2+ oscillations in human esophageal squamous cell carcinoma
Source: Oncotarget. 2014 Apr 17;5(11):3455–71. doi: 10.18632/oncotarget.1903 (PMC4116495; doi:10.18632/oncotarget.1903)
Supplement: Supplementary file 1 [file oncotarget-05-3455-s001.pdf]

Elevated Orai1 expression mediates tumor-promoting intracellular Ca2+ oscillations in human esophageal squamous cell carcinoma

Supplementary Materials

Additional materials and methods

Real-time RT-PCR

Total RNA was extracted from samples using the RNeasy Mini Kit (QIAGEN, MD) following the manufacturer's protocol. Reverse transcription of the RNA was performed according to the protocol included in the TaqMan Reverse Transcription Reagents kit (Roche). The designed primers for real-time RT-PCR were: *orai 1* forward "5'- tcttcctagctgaggtggt -3'", *orai1* reverse "5'- cgaagacgataaagatcagg -3'"; *stim1* forward "5'- agtccgtaacatccacaaac -3'" and *stim1* reverse "5'- acttctgatgacttccatgc -3'"; *β-actin* forward "5'- cagcacaatgaagatcaaga -3'" and *β-actin* reverse "5'- aaagggtgtaacgcaactaa -3'". SYBR Green PCR Master Mix (Applied Biosystems) was used and the amplification cycle was set as 30. All PCR products were verified by agarose gel chromatography (1.5%) to be single bands with the predicted molecular size. All experiments utilizing qRT-PCR were performed at least twice and with all sampling performed in triplicate. C(t) value was calculated with Opticon Monitor software version 1.08 (Bio-Rad) and the expression of mRNA was normalized with housekeeping gene *β-actin.*

Antibodies and other reagents

Additional antibodies used in this study included the following: anti-Tubulin pAb (Abcam, against human 1-100a.a., MA); mouse anti–ß-Actin (Sigma, MO); anti-ERK (Cell Signaling #4695, MA), anti-phosphorylated ERK (Cell Signaling #4370, MA); anti-AKT (1:2000, Cell Signaling #9272, MA), anti-phosphorylated AKT(T308) (Cell Signaling #9275, MA); anti-NCX3 (Santa Cruz, sc-48896, CA), anti-SERCA2 (Santa Cruz, sc-8095, CA); anti-Caveolin-1 (Cell Signaling, MA); anti-STIM2 pAb (ProSci, CA); anti-MEF2D pAb (Bethyl Laboratory, TX). siRNA against STIM1 was from Santa Cruz (sc-76589, CA).

Measurement of basal Ca^{2+} level, ER Ca^{2+} stores and SOCE rate in fura-2 loaded HET-1A and ESCC cells

The fluorescence ratio (F_{350}/F_{385}) at resting stage was used to represent basal Ca^{2+} level. Addition of 10 μM TG in Ca^{2+}-free BSS solution depleted ER Ca^{2+} stores and the difference between basal and maximal values of F_{350}/F_{385} with TG was used to represent ER Ca^{2+} stores ($\Delta F_{350}/F_{385}$). Both basal Ca^{2+} level, ER Ca^{2+} stores were average of at least 50 cells in PMT mode. The influx rates for each individual cell were measured from the slope of the fluorescence ratio (F_{350}/F_{385}) 5-10 s after Ca^{2+} readdition, corrected by its size and represented in the unit of a.u. The SOCE rate was done in cooled-CCD image mode.

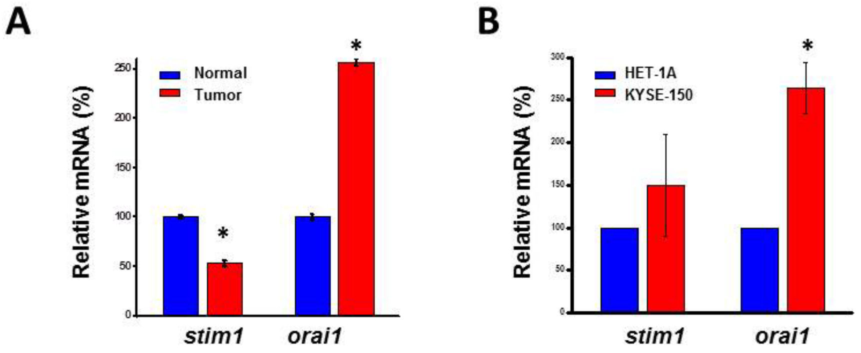

Figure S1: Quantitative real-time RT-PCR of *stim1* and *orai1* genes in tumor tissues removed from patients with ESCC (A) and ESCC cell lines (B). A, mRNA levels of two genes in tumor specimens (red bar) and paired neighboring non-tumorous tissues (blue bar). The results were from total 12 paired samples. B, mRNA levels of two genes in HET-1A (blue bar) and KYSE-150 (red bar) cell lines. The C(t) value was calculated with Opticon Monitor software version 1.08 (Bio-Rad) and the expression of mRNA was normalized with housekeeping gene *β-actin* first and then compared to mRNA in non-tumorous tissues. All experiments have been performed at least twice and each time with triplicated samples. *p<0.01.

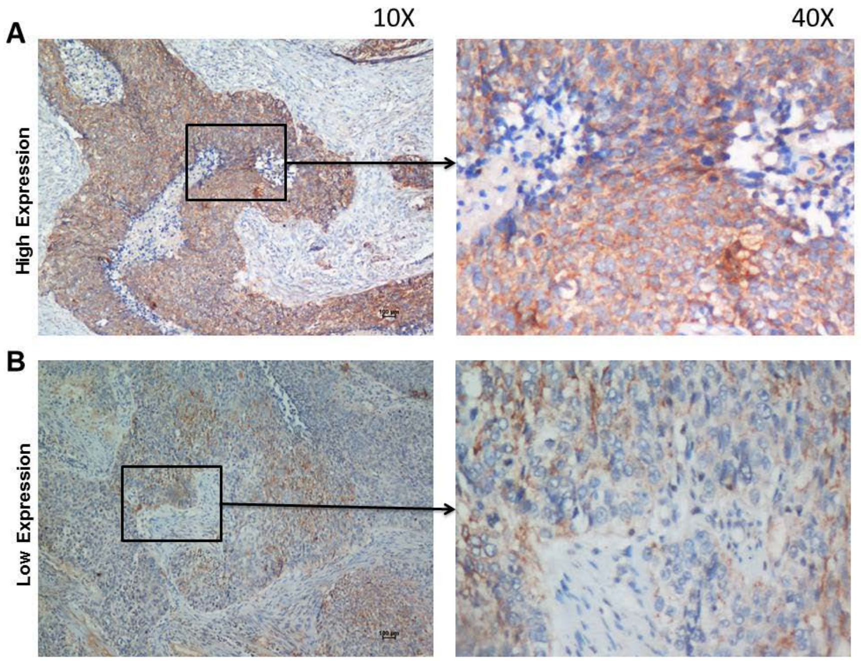

Figure S2: Additional representative images of Orai1 IHC staining in tumor tissues removed from patients with ESCC. A, majority of specimens showed strong membrane staining of Orai1 in tumors. B, some samples demonstrated weak staining of Orai1. Images were taken at different magnification: 10x (left panels) and 40x (right panels).

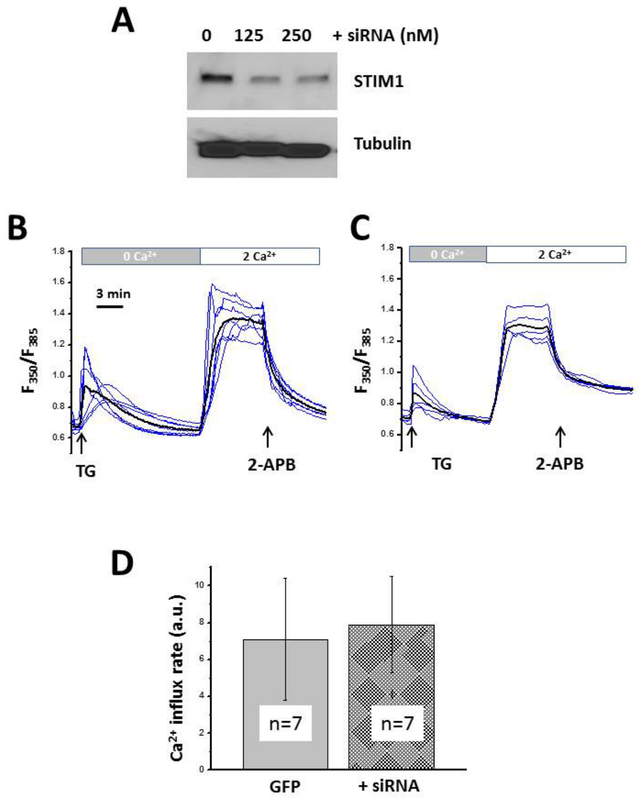

Figure S3: Knockdown of STIM1 had no effect on SOCE in KYSE-150 cells. A, Western blot of STIM1 in KYSE-150 cells transfected with siRNA against STIM1. B and C, intracellular Ca^{2+} concentrations for Fura 2-loaded KYSE-150 cells transfected with plasmids containing GFP (B) or siRNA against STIM1 (C) as a function of treatment with thapsigargin (TG, 5 μM) and EGTA (0.5mM), or with CaCl2 (2 mM). Addition of 2-APB (70 μM) blocked a significant portion of Ca^{2+} influx. D, statistical evaluation of SOCE influx rate in KYSE-150 cells transfected with plasmids containing control (GFP) or siRNA against STIM1 (+siRNA). There was no difference between the two groups.

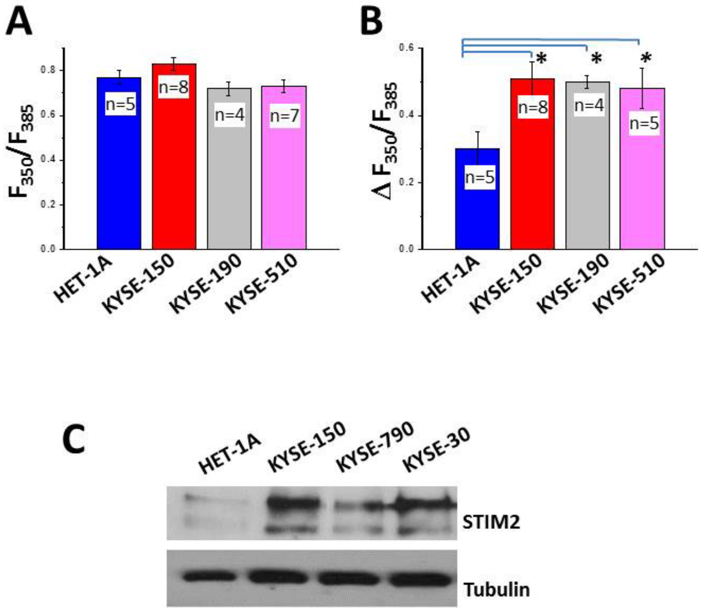

Figure S4: Basal cytosolic and ER Ca^{2+} stores and STIM2 expression in HET-1A and ESCC cells. A, comparison of basal Ca^{2+} levels in HET-1A and ESCC cells. B, comparison of TG releasable ER Ca^{2+} stores in HET-1A and ESCC cells. *p<0.01. C, western blot findings for STIM2 in HET-1A and ESCC cells. The expression level of STIM2 was significantly higher in ESCC cell lines than that in HET-1A cells.

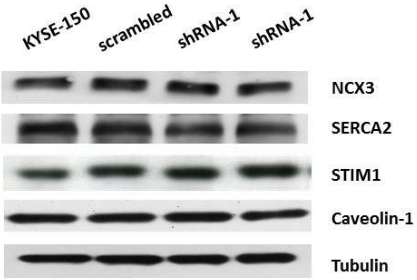

Figure S5: Western blot findings for Ca^{2+} signaling related proteins in ESCC cells. Sodium-calcium exchanger 3 (NCX3), SR/ER Ca^{2+} ATPase 2 (SERCA2), Caveolin-1 and STIM1 were examines in KYSE-150 cells transfected with plasmids containing scrambled or anti-Orai1 shRNAs.

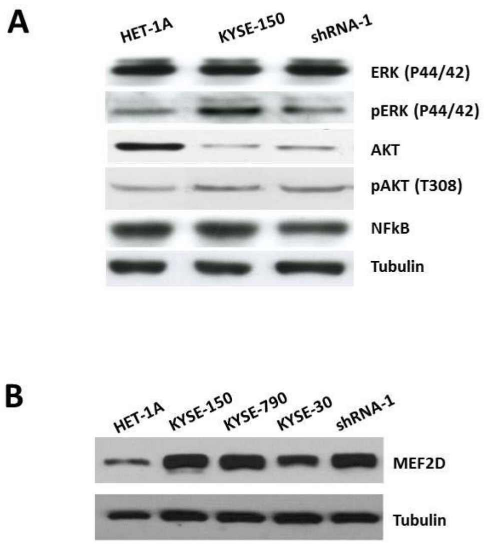

Figure S6: Western blot of several signaling proteins involved in cell proliferation in ESCC cells. ERK, AKT, NFκB (A) and MEF2D (B) were examined.

Table S1: **Summary of IHC staining of Orai1 in ESCC specimens with matched pairs of tumor and neighboring tissues.**

	Tumor Tissue **H**	Tumor Tissue **L**	Total
Neighboring Tissue **H** H	5	0	5
Neighboring Tissue **L**	12	6	18
Total	17	6	23

McNemar Test. $p<0.001$. **H,** high expression; **L,** low expression.

Table S2: Univariate analysis of the factors that influence the prognosis of patients with ESCC.

Variables	Number	Overall Survival			Disease-Free Survial		
		Mean	Median	*p*-value	Mean	Median	*p*-value
Age							
<60 years	39	35.2	30	P=0.499	29.564	21	0.990
≥60 years	43	32.8	22		30.326	21	
Gender							
Male	62	33.8	23	P=0.955	31.339	21	0.405
Female	20	34.2	30		25.950	20	
Grade[a]							
G1	28	40.6	23	P=0.208	37.786	21	0.144
G2	29	31.2	30		26.310	22	
G3	25	29.6	21		25.640	17	
Stage							
I	14	47.500	34	P=0.012	41.286	21	0.026
II	30	36.667	27		33.400	22	
III	38	26.763	20		23.211	16	
TNM classification							
T							
T1/T2	23	43.826	34	P=0.029	39.435	21	0.034
T3/T4	59	30.068	22		26.356	21	
N							
N0	38	41.901	33	P=0.009	37.368	27	0.010
N1/2/3	44	30.144	20		23.773	15	
Orai1 expression							
high	52	25.528	20	p<0.001	20.612	13	<0.001
low	30	48.679	43		46.541	42	
Relapse							
No	26	36.437	27	P=0.132	35.125	27	0.003
Yes	56	28.719	21		19.192	13	

Kaplan-Meier curves with univariate analyses (log-rank); a, Histologic grade is defined as G1 versus G2 versus G3. TNM stage classification is according to AJCC.

Table S3: High Expression of Orai1 with higher relative risk in ESCC patients.

Variables	Overall Survival			Recurrence-free Survival		
	RR	95% CI	*p*-value	RR	95% CI	*p*-value
TNM classification: N	1.647	1.026-2.642	0.039	1.604	1.008-2.552	0.046
Orai1 High expression	2.683	1.620-4.444	<0.001	2.752	1.658-4.567	<0.001

Cox Regression Model. CI, confidence interval; RR, relative risk.
